# Anticipatory prediction in older readers

**DOI:** 10.3758/s13421-025-01712-1

**Published:** 2025-05-06

**Authors:** Roslyn Wong, Aaron Veldre

**Affiliations:** 1 School of Psychology, The University of Sydney, Sydney, Australia; 2 School of Psychological Sciences, Macquarie University, Sydney, Australia; 3 Graduate School of Health, University of Technology Sydney, Sydney, Australia

**Keywords:** Prediction, Reading, Aging, Eye movements, Self-paced reading

## Abstract

It is well-established that skilled, young-adult readers rely on predictive processing during online language comprehension; however, fewer studies have investigated whether this extends to healthy, older adults (60 + years). The aim of the present research was to assess whether older readers make use of lexical prediction by investigating whether they demonstrate processing costs for incorrect predictions in a controlled experimental design. The eye movements of a sample of older adults (60–86 years) were recorded as they read strongly and weakly constraining sentences containing a predictable word or an unpredictable alternative that was either semantically related or unrelated. To determine whether predictive processing depends on the stimuli presentation format, a second experiment presented the same materials in a self-paced reading task in which each word of a sentence appears one at a time at the readers’ own pace. Older adults showed processing benefits for expected input on eye-movement measures of reading. They also showed processing costs for unexpected input across both methodologies, but only when semantically unrelated to the best completion. Taken together, the results suggest that the use of predictive processes remains relatively preserved with age. The implications of these findings for understanding whether prediction is a fundamental component of online language comprehension are discussed.

**Supplementary Information:**

The online version contains supplementary material available at 10.3758/s13421-025-01712-1.

The notion that human comprehenders make use of prediction is widely accepted in the language comprehension literature (Ferreira & Chantavarin, 2018; Huettig, 2015; Kuperberg & Jaeger, 2016; Pickering & Gambi, 2018; Ryskin & Nieuwland, 2023; Wong et al., 2024a). Upcoming words that can be predicted in advance of their presentation require less time and cognitive resources to identify when encountered, allowing for faster and more efficient language processing. In eye-movement studies, words that are predictable in a sentence context receive shorter fixation durations, higher skipping rates, and fewer regressions compared with unpredictable words (see Staub, 2015, for a review). In event-related potential (ERP) studies, predictable words are associated with a reduced N400 component, which captures decreased neural processes for semantically congruent information within a sentence context (see Kutas et al., 2011; Van Petten & Luka, 2012, for reviews). Yet much of what is known about predictive processes is based on data from skilled, young-adult readers. Comparatively fewer studies using these methodologies have investigated prediction in other populations including healthy, older adults (60 + years). It is important to understand if and how predictive processes change across the lifespan and, as such, whether prediction is truly a fundamental component of online language comprehension, in line with more general predictive accounts of cognitive functioning (Clark, 2013; Friston, 2010).

Normal aging is accompanied by widespread neural changes, including grey and white matter atrophy, synaptic degeneration, and neurochemical alterations. This has consequences for cognitive functioning (Cabeza et al., 2004; Hedden & Gabrieli, 2004), including reduced processing speed, attention and executive control, and working memory capacity (see Verhaeghen, 2013, for a review). But not all aspects of cognitive functioning decline with age—language-related functions remain stable, or even improve, especially those that depend on “crystallized” abilities which augment with age and experience. Compared with younger adults, older adults retain higher levels of vocabulary (Alwin & McCammon, 2001; Verhaeghen, 2003) and word-related knowledge (Salthouse, 1993), and organize information in semantic memory as efficiently when assessed via word associations (Bowles et al., 1983; Burke & Peters, 1986) and semantic priming tasks (Laver & Burke, 1993).

However, real-time language comprehension is complex and multifaceted—readers must extract lexical information from the written text, retrieve their meanings from long-term memory, and integrate this information into the unfolding discourse representation. This process relies on the coordination of preserved crystallized abilities like word and semantic knowledge, and age-dependent fluid abilities like working memory and attentional control. Indeed, eye-movement studies show that older adults read more slowly than younger adults as a result of more, and often longer, fixations and more regressions (see Paterson et al., 2020, for a review). Older readers of alphabetic languages also skip words more frequently and make longer forward saccades (Laubrock et al., 2006; Rayner et al., 2006). One influential account put forward to explain this age-related change in eye-movement control is the *risky reading hypothesis*, according to which older adults’ declines in visual and cognitive abilities are offset by stronger reliance on contextual information (Rayner et al., 2006). Whether normal aging has specific consequences for the use of predictive processes, as would be expected under this hypothesis, remains less clear.

## Age-related predictability effects in eye-movement studies

Eye-movement studies indicate at least equivalent effects of predictability during online processing in older and younger adults. This was recently demonstrated in a meta-analysis by Zhang et al. (2022), which found that predictability effects in English did not differ significantly as a function of age, although relatively few studies were analyzed (*n* = 3 for predictability effects on word skipping; Cheimariou et al., 2021; Choi et al., 2017; Rayner et al., 2006). Indeed, some eye-movement studies report *increased* predictability effects on early and late reading measures in older adults (Cheimariou et al., 2021; Choi et al., 2017; Veldre et al., 2022b), suggesting greater use of contextual information with age. However, because these predictability benefits have typically been observed *on* measures of target word processing, older adults may be facilitated in their processing of predictable words not only because these items are easier to *predict* in advance of their presentation but because they are also easier to *integrate* into the unfolding discourse representation (Ferreira & Chantavarin, 2018; Pickering & Gambi, 2018). In practice, disentangling prediction and integration accounts of predictability effects is difficult because both expect facilitated processing for words that can be predicted from prior context.

One way that researchers have attempted to investigate the process of prediction, independently of integration, is by looking at whether there are immediate processing *costs* when readers encounter input that is plausible but unexpected. For example, consider strong and weak contexts like “*The shepherd spent all day looking for his lost...”* and “*The farmer reported that some...*”, respectively, completed by the predictable target for the strong context (“*sheep*”) or by an unpredictable target that is either semantically related (“*cows*”) or unrelated (“*tools*”) to the best completion. Because both unpredictable targets are matched on 0% cloze probability across the constraint conditions, any additional processing for these words in strong versus weak contexts reflects the consequences of violating the expected, but never presented, completion. These effects are likely to reflect prediction processes if they occur on first-pass reading measures, which are assumed to index early lexical processing compared with integration processes, which are captured by later reading measures (Clifton et al., 2007; Vasishth et al., 2013). Using this paradigm, Frisson et al. (2017) found no observable consequences of prediction failure in young adults (see also Wong et al., 2024b). However, when using a larger number of critical items, Wong et al. (2024c) found a small but significant cost on gaze duration for unrelated words in strong contexts (see also Cevoli et al., 2022, for similar evidence from corpus data). Moreover, in both studies, related words actually received *shorter* total reading times and *fewer* regressions-out in strong contexts, suggesting that young adults made graded predictions about upcoming text involving the passive activation of broader morphosyntactic, syntactic, and semantic information (see also Andrews et al., 2022; Luke & Christianson, 2016).

From this perspective, it is plausible that if older adults show preserved, if not enhanced, use of contextual information during reading, they may reveal immediate processing costs when their expectations turn out to be incorrect. Only one eye-movement study by Andrews et al. (2022) has investigated this issue by comparing older and younger adults’ reading of naturalistic texts from the Provo Corpus (Luke & Christianson, 2017). They found that, although reading times for both groups were significantly predicted by cloze probability, neither age group showed prediction error costs because unexpected words received facilitated processing even as the cloze probability of the best completion increased. That is, while older adults do rely on predictive processes, they appear to generate multiple possible continuations for upcoming text, which are less likely to incur processing costs when disconfirmed by unexpected input compared with the preactivation of a single lexical candidate. Notably, however, only 5% of the words in the Provo Corpus are highly predictable (> 0.67 cloze probability), which may have limited the possibility of observing prediction error costs. More generally, corpus data may not be ideal for assessing such effects because naturalistic texts can vary on multiple uncontrolled dimensions which may interact with or obscure the effects of interest (Angele et al., 2015). Thus, it is important to investigate older adults’ processing of unexpected input using a controlled experimental design (see, e.g., Steen-Baker et al., 2017).

## Age-related predictability effects in ERP studies

A further motivation for investigating age-related predictability effects is that ERP studies suggest that older adults are *less* sensitive to contextual information during reading. The late frontal positivity is a positive-going ERP component observed in young adults approximately 500–1,000 ms after unexpected input, typically in strong contexts that encourage the preactivation of a more expected competitor (see Van Petten & Luka, 2012, for a review). Although its precise functional role is debated, this waveform may reflect the additional neural activity required to suppress the more expected completion (Federmeier et al., 2007; Kutas, 1993) and/or revise an existing discourse representation so that the unexpected completion can be integrated successfully (Brothers et al., 2015; DeLong et al., 2014). Older adults, however, do not show this late frontal positivity when processing unexpected input (Federmeier et al., 2010; Wlotko et al., 2012; but see Dave et al., 2018), which has been taken to suggest that predictive processes become less important with age because of general declines in executive control and working memory that impact the coordination of these higher-order processes (see Wlotko et al., 2010, for a review). Further evidence that aging compromises the effective use of top-down contextual information comes from the N400 component—an ERP waveform that is attenuated for words constrained by prior context (see Kutas et al., 2011; Van Petten & Luka, 2012, for reviews)—which is also consistently smaller in amplitude and/or delayed in latency for older adults (see Payne & Silcox, 2019, for a review). In contrast to eye-movement findings then, ERP studies provide little evidence that older adults use prediction during online processing.

However, there are reasons to question whether ERP findings generalize to normal reading. ERP studies typically use a rapid serial visual presentation (RSVP) paradigm in which words appear one at a time at a fixed pace ranging from 400 to 1,000 ms. While the slow word-by-word presentation format may allow for increased strategic prediction (Dambacher et al., 2012; Wlotko & Federmeier, 2015), participants are simultaneously unable to engage in normal reading behavior including skipping words, making regressions to previous parts of text, and processing upcoming words in the parafovea. The RSVP paradigm may therefore engage different online processes to normal reading, raising the possibility that evidence of age-related declines in prediction could be restricted to tasks involving unnatural presentation formats.

## Another approach to investigating age-related predictability effects

One way to provide insight into whether predictive processes in older adults depend on stimuli presentation method is the self-paced reading (SPR) paradigm in which each word of a sentence appears one at a time at the readers’ own pace. Although this methodology uses a word-by-word presentation format, it does simulate normal reading more closely by allowing readers control over the presentation rate. For instance, Payne and Federmeier (2017) found that young adults who could self-pace their reading produced the late frontal positivity for prediction violations on trials where they elicited faster response times, roughly equivalent to the fixed-pace presentation rate used in RSVP studies (~ 2 words per second). On trials with slower response times, young adults produced an anterior N2, a negative-going component approximately 200–350 ms poststimulus onset that was taken to index a motor inhibitory signal to slow down reading and engage in conflict resolution (see Folstein & Van Petten, 2008, for a review). Thus, volitional control over input rate appears to play a role in modulating readers’ sensitivity to contextual information. Investigating how older adults use contextual information during self-paced reading will contribute to the broader questions of whether age-related predictive processes are modulated by stimuli presentation method and whether the outcomes of self-paced reading are comparable to that of normal reading.

## The present research

The present research aimed to investigate older adults’ use of prediction during reading. The primary goal was to establish whether older adults show evidence of processing costs for incorrect predictions in a controlled experimental design. Thus, Experiment 1 recorded older adults’ eye movements as they read strongly and weakly constraining sentences containing predictable and unpredictable words in a natural reading task. A further goal was to assess whether older adults’ predictive processes depend on stimuli presentation method. Thus, Experiment 2 recorded a separate sample of older adults’ reading times as they read the same sentence materials in a SPR task in which each word of a sentence appears one at a time at the readers’ own pace. The pattern of predictability benefits and/or costs across the two methodologies may help reconcile the discrepant conclusions from eye-movement and ERP studies regarding the impact of aging on prediction during reading.

## Experiment 1

Older adults’ eye movements were recorded as they read sentences that were either strongly or weakly constraining towards a specific word. The target word presented was either the predictable word for the strong context or an unpredictable word. To assess the preactivated information, unpredictable words were either semantically related or unrelated to the best completion. If older adults use prediction, predictable words in strong contexts should yield larger processing benefits relative to the same words in weak contexts. If these predictions involve preactivating semantic features of upcoming words, rather than a specific lexical candidate, these processing benefits should extend to related words in strong contexts. If older adults do make predictions about upcoming text, there should also be evidence of immediate processing costs when their expectations turn out to be incorrect. That is, unrelated words should be processed less efficiently on early reading measures in strong versus weak contexts.

### Method

#### Transparency and openness

The experimental materials, de-identified data, and analysis codes for both experiments are publicly available (https://osf.io/w6594/). This research was approved by the University of Sydney Human Research Ethics Committee (Project title: What do you expect when you read? Project number: 2019/180). This study was not preregistered.

#### Participants

Fifty healthy, cognitively intact older adults living independently in the urban community participated in return for cash reimbursement. Six participants’ data were excluded due to eye-tracking calibration difficulty and/or self-reported visual impairments. The final sample comprised of 44 participants (*M*_age_ = 70.5 years, range: 60–86 years, 32 women). Most older adults had completed some form of post-secondary education and 78% had a college degree. All older adults performed above average on the National Adult Reading Test (Nelson, 1982), which has been shown to be highly correlated with the WAIS-IV (Bright et al., 2018), and within the normal range for their age and education level on tests of phonemic and semantic verbal fluency. All participants were native English speakers, and their corrected visual acuity was assessed by a modified Snellen test at the experimental viewing distance to be better than 20/40.

#### Materials

The stimuli were 66 pairs of sentences from Wong et al. (2024c). Each pair comprised a strong context sentence, in which the target word was highly predictable, and a weak context sentence, in which the same target word was not predictable. The predictable target was compared with length- and frequency-matched unpredictable targets that were either semantically related or unrelated. To ensure that predictable targets in strong contexts were predictable (> 0.5) and targets in other conditions were unpredictable (< 0.2), a separate sample of 20 participants (*M*_age_ = 20.4 years; 19 women) provided cloze completions. To ensure that related and unrelated targets were equivalently plausible across conditions, another sample of 60 participants (*M*_age_ = 19.7 years; 49 women) judged the sentences up to and including the target on a 5-point scale from 1 (*Highly Implausible*) to 5 (*Highly Plausible*). Target semantic relatedness was assessed by computing Latent Semantic Analysis (LSA; Landauer & Dumais, 1997) scores between predictable targets and each unpredictable target. Table 1 presents an example item with mean lexical characteristics by condition.

**Table 1:** Example set of items and mean (and standard deviation) stimulus characteristics

Condition	Example item (target bolded)	Target cloze probability		Target frequency (logHAL)	Target length (letters)	Sentence plausibility (1–5 scale)	Target relatedness to predictable word (LSA)	Sentence constraint
Strong constraint								
Predictable	The shepherd spent all day looking for his lost **sheep** in the fields despite the rain.	.84 (.12)		9.50 (1.45)	5.4 (1.3)	4.9 (0.1)	1 (0)	0.84 (0.12)
Related	The shepherd spent all day looking for his lost **cows** in the fields despite the rain.	.01 (.03)		8.73 (1.92)	5.4 (1.4)	4.8 (0.4)	0.35 (0.21)	0.84 (0.12)
Unrelated	The shepherd spent all day looking for his lost **tools** in the fields despite the rain.	.00 (.01)		9.32 (1.81)	5.4 (1.3)	4.5 (0.4)	0.15 (0.12)	0.84 (0.12)
Weak constraint								
Predictable	The farmer reported that some **sheep** had been stolen from his property.	.02 (.04)	9.50 (1.45)		5.4 (1.3)	4.9 (0.1)	1 (0)	0.18 (0.07)
Related	The farmer reported that some **cows** had been stolen from his property.	.01 (.02)	8.73 (1.92)		5.4 (1.4)	4.8 (0.3)	0.35 (0.21)	0.18 (0.07)
Unrelated	The farmer reported that some **tools** had been stolen from his property.	.01 (.03)	9.32 (1.81)		5.4 (1.3)	4.7 (0.4)	0.15 (0.12)	0.18 (0.07)

#### Apparatus

The sentences were presented in 14-pt Consolas black font on a white background and displayed on a 21-in. ViewSonic G225f CRT monitor, which was set to a pixel resolution of 1,024 × 768 and a 140 Hz refresh rate. Sentences were either presented as a single line of text or across two double-spaced lines. Target words never appeared at the beginning or end of a line. An SR Research EyeLink 1000 eye-tracker recorded participants’ eye movements at a sampling rate of 1000 Hz. Participants were seated 60 cm from the monitor with a chin and forehead rest to minimize head movements. At this distance, one degree of visual angle equated to 2.85 letter spaces. Viewing was binocular, but eye movements were recorded from participants’ right eye.

#### Procedure

Participants read each sentence for meaning and responded to comprehension questions after approximately one third of the trials. Mean comprehension accuracy was very high (*M* = 94.1%). A 9-point calibration procedure was conducted before the start of the experiment. Maximum calibration error was 0.5° of visual angle. Each trial began when a stable fixation was detected on a fixation point at the location of the sentence’s first letter.

Sentences were counterbalanced across three lists using a Latin square design. Participants always saw a different target word in the strong and weak context version of each pair. Across all sentences, participants saw 22 target words in each of the six conditions. Experimental items were presented in a random order across four blocks interspersed with 26 filler items.

### Results

Fixations below 80 ms were merged with adjacent fixations within one letter space (1.4% of total fixations). Trials were removed if there was track loss or blinks on the target (2.5% of trials). Remaining target fixations below 80 ms or above 800 ms, gaze durations above 1200 ms, and total fixation durations above 2000 ms were excluded (1.7% of trials). These exclusions left 5,562 trials (95.7% of the data) for analysis.

The following log-transformed fixation duration measures on the target were analyzed: *first fixation duration* (the duration of the first fixation on the target), *gaze duration* (the sum of all fixations before the eyes exit the target for the first time), and *total fixation duration* (the sum of all fixations on a target). The probability of skipping, and regressions out of the target to earlier in the sentence, and regressions into the target from later in the sentence were also analyzed. Table 2 presents mean target reading measures by condition.

**Table 2:** Mean (and standard deviation) target reading measures by condition in Experiment 1

	Predictable		Related		Unrelated	
	Strong constraint	Weak constraint	Strong constraint	Weak constraint	Strong constraint	Weak constraint
Skipping (%)	25 (9)	19 (8)	24 (9)	19 (8)	21 (9)	21 (9)
First fixation (ms)	203 (19)	211 (17)	220 (20)	222 (22)	222 (21)	217 (19)
Gaze (ms)	223 (25)	228 (20)	247 (23)	244 (26)	250 (27)	235 (22)
Total fixation (ms)	260 (53)	317 (47)	314 (49)	368 (52)	354 (42)	342 (40)
Regressions-out (%)	11 (6)	14 (8)	12 (8)	14 (7)	14 (10)	13 (8)
Regressions-in (%)	12 (7)	22 (10)	20 (10)	31 (11)	25 (9)	27 (9)

The data were analyzed by (generalized) linear mixed effects models (GLMM/LMM) using R (Version 4.3.1; R Core Team, 2023) and the *lme4* package (Version 1.1–30; Bates et al., 2015). The models tested the fixed effect of constraint (strong vs. weak) nested under target type, consistent with the analyses conducted by Frisson et al. (2017) and Wong et al. (2024c). This returned estimates of the constraint effect separately for predictable, related, and unrelated words, eliminating the possible confound of small differences in lexical characteristics between the target conditions. For predictable words, the constraint effect tested the benefit of making a correct prediction because these words were high cloze in strong contexts but low cloze in weak contexts, while, for unpredictable words, the constraint effect tested the cost of making an incorrect prediction because these words disconfirmed a more expected completion in the strong but not weak contexts. The models also included the main effect of target type coded as a set of two orthogonal contrasts testing the effect of (1) *target predictability*—the difference between the predictable and the average of the related and unrelated conditions, and (2) *target relatedness*—the difference between the related and unrelated conditions. Because these contrasts average over constraint, they are not directly relevant to the interpretation of target word processing; however, their inclusion is important for the purpose of accounting for model variance (Schad et al., 2020). The results below therefore focus only on the outcomes of the constraint effect for each target type, but the complete model output is summarized in Table 3.

**Table 3:** LMM summaries for analyses of target reading measures in Experiment 1

Measure	Fixed effect	*b*	*SE*	*t*/*z*
Skipping	Intercept	** − 1.48**	**0.14**	** − 10.93**
	Predictability	0.02	0.07	0.32
	Relatedness	− 0.00	0.08	− 0.00
	Pred target: Constraint	** − 0.40**	**0.14**	** − 2.86**
	Rel target: Constraint	** − 0.33**	**0.12**	** − 2.64**
	Unrel target: Constraint	− 0.03	0.13	− 0.25
First fixation	Intercept	**5.32**	**0.02**	**217.74**
	Predictability	** − 0.06**	**0.01**	** − 6.31**
	Relatedness	0.00	0.01	0.02
	Pred target: Constraint	**0.04**	**0.02**	**2.16**
	Rel target: Constraint	0.01	0.02	0.26
	Unrel target: Constraint	− 0.01	0.02	− 0.63
Gaze	Intercept	**5.39**	**0.03**	**182.31**
	Predictability	** − 0.08**	**0.01**	** − 7.01**
	Relatedness	0.00	0.01	0.18
	Pred target: Constraint	0.02	0.02	1.02
	Rel target: Constraint	− 0.02	0.03	− 0.75
	Unrel target: Constraint	** − 0.05**	**0.02**	** − 2.05**
Total fixation	Intercept	**5.62**	**0.04**	**136.92**
	Predictability	** − 0.17**	**0.01**	** − 12.00**
	Relatedness	− 0.01	0.02	− 0.86
	Pred target: Constraint	**0.17**	**0.03**	**5.00**
	Rel target: Constraint	**0.13**	**0.04**	**3.43**
	Unrel target: Constraint	− 0.02	0.03	− 0.71
Regressions-out	Intercept	** − 2.14**	**0.14**	** − 14.82**
	Predictability	− 0.08	0.09	− 0.87
	Relatedness	− 0.02	0.10	− 0.19
	Pred target: Constraint	**0.33**	**0.16**	**2.06**
	Rel target: Constraint	0.27	0.16	1.67
	Unrel target: Constraint	− 0.23	0.20	− 1.11
Regressions-in	Intercept	** − 1.38**	**0.11**	** − 12.97**
	Predictability	** − 0.58**	**0.08**	** − 7.39**
	Relatedness	− 0.09	0.08	− 1.07
	Pred target: Constraint	**0.76**	**0.18**	**4.22**
	Rel target: Constraint	**0.68**	**0.18**	**3.70**
	Unrel target: Constraint	0.12	0.17	0.73

*Note. *Significant effects are bolded. Pred = predictable; Rel = related; Unrel = unrelated

All models showed singular fits with the maximal random effects structure (by-subject and by-item random intercepts and slopes for the effect of constraint nested under target type). Therefore, each model’s random effects structure was simplified: first by removing the correlation parameters between random intercepts and random slopes, and second by sequentially removing random slopes that accounted for the lowest variance until model convergence without singular fit. Estimates yielding *t*/*z* values greater than |1.96| were interpreted as significant at the 0.05 α level. Power analyses conducted with 100 Monte Carlo simulations using the *simR* package (Version 1.0–6; Green & MacLeod, 2016) in R demonstrated sufficient power (> 0.80) to detect the constraint effect for each target type of at least 10 ms on first fixation duration, 15 ms on gaze duration, and 31 ms on total fixation duration.

For predictable targets, the effect of constraint was significant on all reading measures (|*t*/*z*|s > 2.06) except gaze duration (*t* = 1.02)—predictable words showed higher skipping rates, shorter first and total fixation durations, and fewer regressions in strong versus weak contexts. For related targets, the effect of constraint was significant on skipping, total fixation duration, and regressions-in (|*t*/*z*|s > 2.64) due to higher skipping rates, shorter total reading times, and fewer regressions-in for these words in strong versus weak contexts. For unrelated targets, the effect of constraint was significant on gaze duration (*t* = − 2.05)—readers had *longer* first-pass reading times when these words were presented in strong versus weak contexts. Thus, predictable completions received early and late processing benefits in strong contexts. Related completions that violated these expectations yielded similar processing benefits in strong contexts. However, there was some limited evidence of a small prediction error cost for unrelated completions—older adults showed disruption during first-pass reading when processing these words in strong contexts.^1^

#### Supplementary analyses

To directly test age-related differences in predictive processes, an additional set of analyses compared the present data with the data of a sample of young adults from Wong et al.’s (2024c) Experiment 2 (*n* = 57, *M*_age_ = 21.2 years, range: 17–32 years, 40 women) who showed evidence of a processing cost for unrelated words on gaze duration. All aspects of Wong et al.’s experiment were the same as the present study, except young adults read the sentence materials as the first sentence of a two-sentence passage and some items differed by a small number of words that did not change the target predictability or meaning of the sentence. The age groups did not differ significantly on mean comprehension scores,* t* < 1. Following previous eye-movement studies (Cheimariou et al., 2021; Choi et al., 2017; Veldre et al., 2022b), older adults were expected to show equivalent, if not larger, effects of predictability compared with younger adults.

**Fig. 1:**
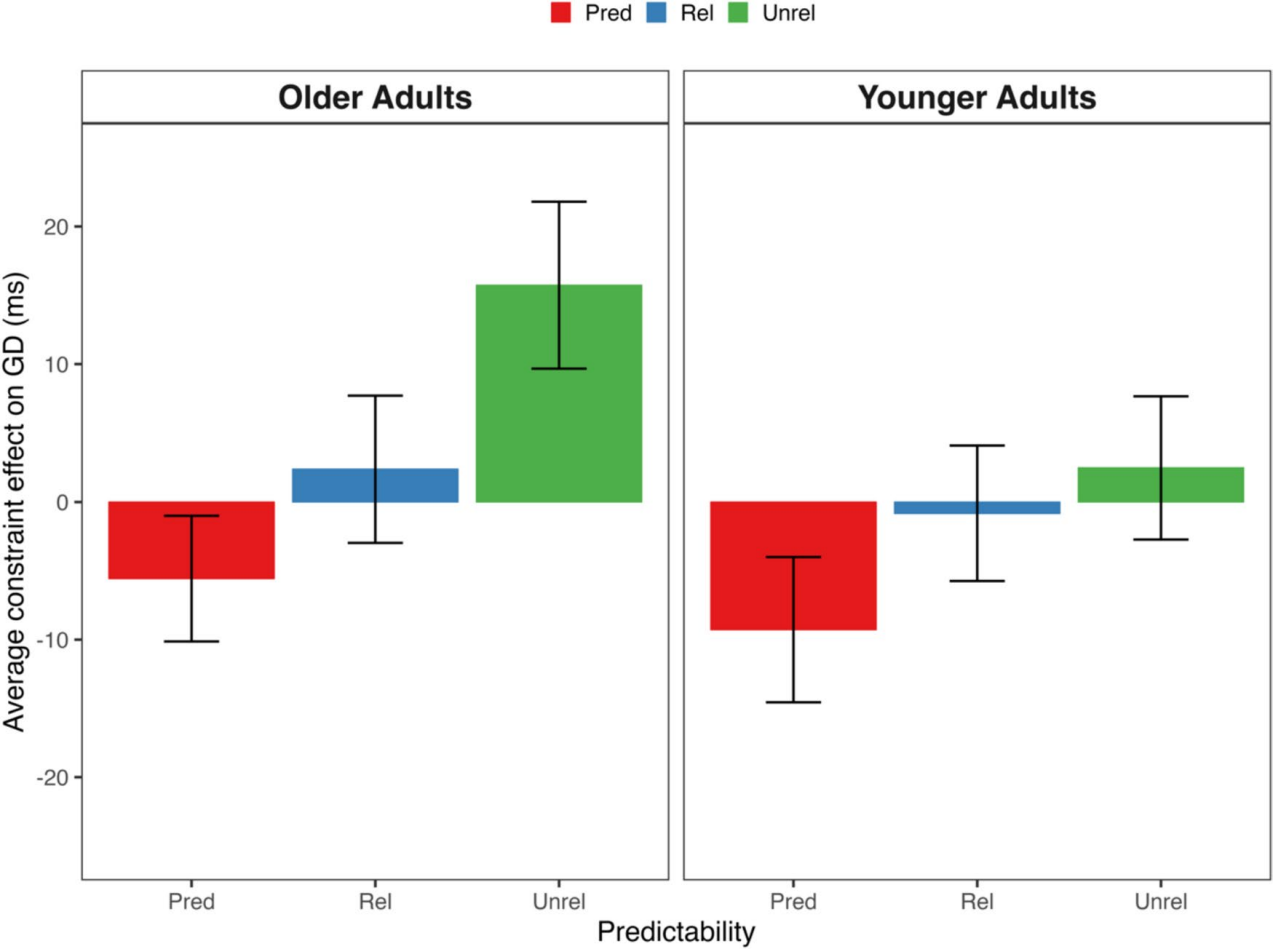
Mean constraint effect on gaze duration across targets for older and young adults in Experiment 1. *Note.* Positive constraint effects reflect longer reading times in strong versus weak contexts. Younger adults (*n* = 57) from Wong et al. (2024c; Experiment 2). (Colour figure online)

The combined analyses compared the following first-pass target reading measures: log-transformed first fixation and gaze duration, and the probability of skipping and regressions-out of the target.^2^ Figure 1 presents older and younger adults’ means by condition on gaze duration, which showed the significant constraint effect for unrelated targets in both age groups. The data were analyzed by (G)LMMs which tested the fixed effect of age, and the fixed effect of constraint nested under target type. Table 4 summarizes the complete model output. Power analyses conducted with 100 Monte Carlo simulations demonstrated sufficient power (> 0.80) to detect Age × Constraint interactions for each target type of at least 14 ms effect size on first fixation duration and 20-ms effect size on gaze duration.

**Table 4:** LMM summaries for analyses of target reading measures for older and younger adults in Experiment 1

Measure	Fixed effect	*b*	*SE*	*t*/*z*
Skipping	Intercept	** − 1.28**	**0.09**	** − 13.53**
	Age	** − 0.41**	**0.16**	** − 2.61**
	Predictability	0.09	0.05	1.85
	Relatedness	− 0.03	0.05	− 0.57
	Pred target: Constraint	** − 0.35**	**0.09**	** − 3.97**
	Rel target: Constraint	** − 0.23**	**0.09**	** − 2.67**
	Unrel target: Constraint	− 0.02	0.08	− 0.28
	Age × Predictability	− 0.13	0.09	− 1.37
	Age × Relatedness	0.05	0.11	0.49
	Age × Pred target: Constraint	− 0.11	0.15	− 0.72
	Age × Rel target: Constraint	− 0.18	0.17	− 1.10
	Age × Unrel target: Constraint	− 0.01	0.15	− 0.09
First fixation	Intercept	**5.32**	**0.01**	**356.37**
	Age	− 0.00	0.03	− 0.07
	Predictability	** − 0.06**	**0.01**	** − 9.37**
	Relatedness	0.01	0.01	0.81
	Pred target: Constraint	**0.03**	**0.01**	**2.21**
	Rel target: Constraint	0.00	0.01	0.35
	Unrel target: Constraint	− 0.01	0.01	− 0.97
	Age × Predictability	− 0.00	0.01	− 0.08
	Age × Relatedness	− 0.01	0.01	− 0.78
	Age × Pred target: Constraint	0.03	0.02	1.21
	Age × Rel target: Constraint	0.00	0.02	0.14
	Age × Unrel target: Constraint	0.00	0.02	0.04
Gaze	Intercept	**5.39**	**0.02**	**300.53**
	Age	− 0.00	0.03	− 0.02
	Predictability	** − 0.08**	**0.01**	** − 10.12**
	Relatedness	0.02	0.01	1.75
	Pred target: Constraint	0.03	0.02	1.81
	Rel target: Constraint	− 0.01	0.02	− 0.52
	Unrel target: Constraint	** − 0.03**	**0.02**	** − 2.09**
	Age × Predictability	0.00	0.01	− 0.01
	Age × Relatedness	− 0.02	0.02	− 1.45
	Age × Pred target: Constraint	− 0.01	0.03	− 0.40
	Age × Rel target: Constraint	− 0.02	0.03	− 0.72
	Age × Unrel target: Constraint	− 0.03	0.03	− 1.10
Regressions-out	Intercept	** − 2.05**	**0.09**	** − 22.02**
	Age	− 0.17	0.17	− 1.02
	Predictability	− 0.06	0.06	− 1.03
	Relatedness	0.03	0.07	0.44
	Pred target: Constraint	**0.45**	**0.10**	**4.37**
	Rel target: Constraint	0.22	0.12	1.82
	Unrel target: Constraint	− 0.10	0.16	− 0.63
	Age × Predictability	0.01	0.12	0.05
	Age × Relatedness	− 0.08	0.13	− 0.60
	Age × Pred target: Constraint	− 0.23	0.19	− 1.22
	Age × Rel target: Constraint	0.09	0.19	0.50
	Age × Unrel target: Constraint	− 0.22	0.23	− 0.97

*Note.* Significant effects are bolded. Younger adults (*n* = 57) from Wong et al. (2024c; Experiment 2). Pred = predictable; Rel = related; Unrel = unrelated

The outcomes of the combined analyses averaged over age were identical to the outcomes of the analyses restricted to older adults. The main effect of age was only significant on skipping rate which was lower for older compared with younger adults (21% vs. 28%; *z* = − 2.61). Age did not interact significantly with the constraint effect for any target type (|*t*/*z*|s < 1.22). Thus, as illustrated in Fig. 1, both age groups showed similar patterns of target predictability benefits and costs, although older readers also showed lower skipping rates which is inconsistent with the risky reading hypothesis (e.g., Paterson et al., 2020; Rayner et al., 2006).^3^

### Discussion

Experiment 1 assessed older adults’ use of anticipatory prediction by investigating whether they show processing costs for incorrect predictions in a natural reading task.

Following previous eye-movement investigations of this age group (e.g., Cheimariou et al., 2021; Choi et al., 2017; Veldre et al., 2022b), older adults showed more efficient processing for predictable words in strong versus weak contexts. These processing benefits emerged on the early measures of first fixation duration and the probability of skipping and regressions-out, which is consistent with the preactivation of these items in advance of their presentation, as well as the late measures of total fixation duration and the probability of regressions-in, which is more compatible with the facilitated integration of these items into the prior context. Older adults also processed unpredictable words that were related to the best completion more efficiently in strong versus weak contexts. These processing benefits were evident on the late measures of total fixation duration and regressions-in, suggesting facilitated integration of words sharing semantic overlap with the most expected completion. Because these processing benefits additionally emerged on skipping rates, older readers may have also partially preactivated these items ahead of time, either due to spreading activation from the most predictable word (Collins & Loftus, 1975; Neely, 1977) or because the context activated multiple possible continuations based on the available semantic information (Andrews et al., 2022; Luke & Christianson, 2016).

Importantly, older adults showed evidence of an early processing cost for unexpected input that disconfirmed their expectations. Unpredictable words that were unrelated to the best completion received an immediate, albeit small (15 ms) and short-lived, processing disadvantage on gaze duration in strong versus weak contexts, suggesting that older readers were sensitive to the mismatch between their expectations and the word actually encountered. Contrary to Andrews et al.’s (2022) findings then, older readers do appear to be sensitive to the consequences of prediction failure. This finding may have been obscured in the previous study because older readers were presented with a corpus of naturalistic texts in which highly predictable words were rare, limiting the opportunity for strong predictions to be disconfirmed.

The present findings thus indicate that predictive processes appear to be relatively preserved across the lifespan. Older adults showed processing benefits for predictable words that extended to semantically related alternatives, similar to a number of previous findings in their younger counterparts (e.g., Frisson et al., 2017; Wong et al., 2024b, 2024c). Older adults also showed a processing cost for unpredictable words that were semantically unrelated to the best completion, consistent with recent observations of a similar cost during first-pass reading in young adults (Cevoli et al., 2022; Wong et al., 2024c; but see Frisson et al., 2017; Luke & Christianson, 2016; Wong et al., 2024b).^4^ The supplementary combined analyses of older and younger adults’ first-pass reading data provided further support for the age-related preservation of predictability effects, revealing that the benefits on predictable and related targets and the costs on unrelated targets appeared to be of similar magnitude across the age groups, despite the small differences in text format. However, the present findings are inconsistent with ERP findings of no prediction error costs in older adults (Federmeier et al., 2010; Wlotko et al., 2012; but see Dave et al., 2018, for evidence under certain task instructions, Federmeier et al., 2002, 2010, for evidence modulated by verbal fluency ability), which could be attributed to the unnatural RSVP paradigm that discourages effective use of context to generate predictions about upcoming text. To provide some insight into the source of this discrepancy, Experiment 2 investigated older adults’ use of anticipatory prediction in a SPR task which uses an unnatural presentation format.

## Experiment 2

Experiment 2 presented the same sentence materials as the previous experiment in a SPR task in which each word appears one at a time at the readers’ own pace. Previous studies investigating the impact of age on self-paced reading have found that older adults are more disrupted by the unnatural presentation format, but they do show enhanced sensitivity to contextual information compared with their younger counterparts. For example, Stine-Morrow et al. (1996, 2008) observed greater contextual facilitation in older compared with younger adults when the presence of accumulating contextual constraint reduced reading times during text processing. Miller et al. (2006) similarly reported larger contextual benefits with age when readers processed passages accompanied by contextually relevant titles compared with passages without them. Notably, however, no existing research has directly manipulated word predictability to assess whether contextual information is used in a predictive manner during self-paced reading.

If older adults use prediction despite the word-by-word presentation format, they should show patterns of predictability effects similar to Experiment 1. However, if older adults do not make predictions about upcoming text because of the word-by-word presentation format, predictable words should be processed equivalently across strong versus weak contexts, and this should extend to related words. Moreover, unrelated words should yield no processing costs in strong versus weak contexts given that no other lexical candidates would have been expected and subsequently disconfirmed. Different patterns of target word processing compared with the previous experiment may suggest that the source of older adults’ weaker prediction in ERP studies is the overall unnatural presentation format.

### Method

#### Participants

Thirty-nine healthy, cognitively intact older adults (*M*_age_ = 73.5 years, range: 62–89 years, 28 women) living independently in the urban community participated in return for cash reimbursement.^5^ None of the participants had completed Experiment 1. Most older adults had completed some form of postsecondary education and 69% had a college degree. All older adults performed within the normal range for their age and education level on tests of phonemic and semantic verbal fluency. All participants were native English speakers and had self-reported corrected-to-normal vision.

#### Materials

The critical stimuli were the same sentences as in Experiment 1.

#### Procedure

The SPR task was implemented in JavaScript using the *jspysch* library (de Leeuw, 2015) to allow participants to complete the task in a web browser on their own desktop or laptop device due to restrictions on laboratory-based data collection during the COVID-19 pandemic. Participants were instructed to read the sentences for meaning and to respond to comprehension questions which appeared after approximately a third of the trials. Mean comprehension accuracy was very high (*M* = 95.7%).

Each trial began with a fixation cross shown centrally for 1,000 ms before the sentence frame was presented with all nonspace characters replaced by underscores. Participants pressed the space bar to view each word of the sentence, and progressing to the next word replaced the previous word with an underscore. After three practice trials, participants were randomly assigned to one of three lists which were counterbalanced like Experiment 1. Following the task, participants were given the opportunity to report any technical difficulties experienced during the task.

### Results

Reading times were analyzed for the target word of each sentence. To capture possible spill-over effects (Mitchell, 1984), reading times were also analyzed for the word following the target (*Target* + 1). To confirm that effects at the target reflected manipulations of this word, reading times were also analyzed for the word preceding the target (*Target *– 1). Trials were excluded if data were missing (0.8% of trials) or reflected outlier reading times on any of the three interest regions (< 80 ms or > 5,000 ms; 0.05% of the data). Following Payne and Federmeier (2017), reading times on the three interest regions that were more than three standard deviations above a participants’ condition mean were also removed (1.7% of the data). These exclusions left 5,030 Target − 1 data points (97.7% of the data), 5,029 target data points (97.7% of the data), and 5,015 Target + 1 data points (97.5% of the data) for analysis. Reading times were log-transformed for the three interest regions. Table 5 presents mean reading measures by condition and region.

**Table 5:** Mean (and standard deviation) reading times on the interest regions by condition in Experiment 2

	Predictable		Related		Unrelated	
	Strong constraint	Weak constraint	Strong constraint	Weak constraint	Strong constraint	Weak constraint
Target − 1	495 (153)	483 (144)	487 (155)	485 (137)	497 (162)	484 (156)
Target	512 (181)	525 (201)	528 (220)	516 (184)	547 (246)	507 (188)
Target + 1	498 (158)	504 (149)	525 (189)	514 (156)	556 (211)	504 (161)

The data were analyzed by LMMs which tested the same fixed and random effects as Experiment 1. To control for differences in the words before and after the target, the models for these two interest regions also included word length and log HAL frequency as centered, continuous predictors (Lund & Burgess, 1996). Like Experiment 1, the results below focus on the outcomes of the constraint effect for each target type, but the complete model output is summarized in Table 6. Criteria for the random effects structures and significance thresholds were identical to Experiment 1. Power analyses conducted with 100 Monte Carlo simulations demonstrated sufficient power (> 0.80) to detect the constraint effect for each target type of at least 25 ms at each interest region.

**Table 6:** LMM summaries for analyses of reading times on the interest regions in Experiment 2

Measure	Fixed effect	*b*	*SE*	*t*
Target − 1	Intercept	**6.13**	**0.04**	**138.68**
	Predictability	0.00	0.01	0.52
	Relatedness	− 0.00	0.01	− 0.17
	Length	**0.01**	**0.00**	**2.65**
	Frequency	− 0.00	0.00	− 1.68
	Pred target: Constraint	− 0.01	0.01	− 0.72
	Rel target: Constraint	0.01	0.01	0.59
	Unrel target: Constraint	− 0.01	0.01	− 1.04
Target	Intercept	**6.17**	**0.05**	**119.70**
	Predictability	− 0.01	0.01	− 0.83
	Relatedness	− 0.00	0.01	− 0.49
	Pred target: Constraint	0.01	0.02	0.88
	Rel target: Constraint	− 0.01	0.02	− 0.48
	Unrel target: Constraint	** − 0.05**	**0.02**	** − 2.68**
Target + 1	Intercept	**6.18**	**0.05**	**133.79**
	Predictability	** − 0.03**	**0.01**	** − 4.92**
	Relatedness	− 0.01	0.01	− 1.56
	Length	**0.01**	**0.00**	**2.75**
	Frequency	0.00	0.00	0.42
	Pred target: Constraint	0.02	0.01	1.57
	Rel target: Constraint	− 0.01	0.02	− 0.95
	Unrel target: Constraint	** − 0.07**	**0.02**	** − 3.44**

*Note. *Significant effects are bolded. Pred = predictable; Rel = related; Unrel = unrelated

#### Target − 1

There was no constraint effect on reading times at the word preceding any target type (|*t*|s < 1.04).

#### Target

For both predictable and related targets, the constraint effect was not significant on target reading times (|*t*|s < 1), suggesting that these words were processed equivalently in strong versus weak contexts. For unrelated targets, there was a significant effect of constraint (*t* = − 2.68) because these words received *longer* reading times in strong versus weak contexts (i.e., prediction error cost). Thus, while there were no processing benefits for predictable or related completions in strong contexts, there was evidence of a processing cost when an unrelated completion appeared instead.

#### Target + 1

For both predictable and related targets, the constraint effect was not significant on reading times at the word following the target (|*t*|s < 1.56) because processing effort was equivalent for these targets in strong versus weak contexts. For unrelated targets, there was a significant effect of constraint (*t* = − 3.44) because reading times were longer following these words in strong versus weak contexts. Thus, the spill-over region showed no processing benefits for either predictable or related completions, but a processing cost following unrelated completions in strong contexts.

#### Supplementary analyses

To provide insight into age-related differences in predictive processes, an additional set of analyses compared the present data to the data of a sample of 67 young adults (*M*_age_ = 19.70 years; range: 17–31; 44 women) who also completed the SPR task. All aspects of this experiment were the same except young adults read the sentence materials as part of a two-sentence passage and some items differed by a small number of words that did not change the target predictability or meaning of the sentence. One quarter of the two-sentence passages also included an unpredictable target that was semantically and syntactically anomalous within the first sentence. Mean comprehension accuracy was very high (*M* = 92.3%), indicating that young adults were reading for comprehension, but significantly lower than the sample of older adults, *t*(104) = 3.45, *p* < .001.

**Fig. 2:**
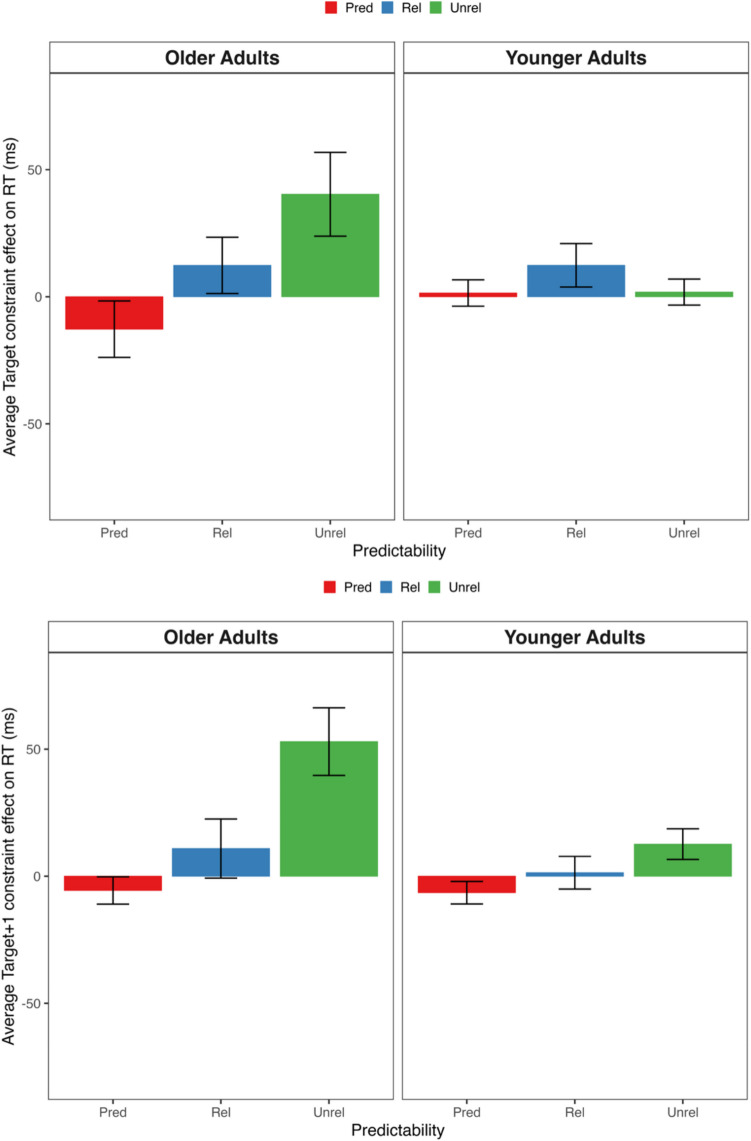
Mean constraint effect on reading times for the target (upper panel) and the word following the target (lower panel) across conditions for older and younger readers in Experiment 2. *Note.* Positive constraint effects reflect longer reading times in strong versus weak context. (Colour figure online)

The combined analyses compared log-transformed SPR times at the three interest regions. Figure 2 presents older and young adults’ mean reading times by condition on the target and the word following the target. LMMs included the same fixed and random effects as Experiment 1’s combined target analyses, except models for the words before and after the target also included word length and log HAL frequency as centered, continuous predictors. Table 7 summarizes the complete model output. Power analyses conducted with 100 Monte Carlo simulations demonstrated sufficient power (> 0.80) to detect Age × Constraint interaction effects for each target type of at least 27 ms at each interest region.

**Table 7:** LMM summaries for analyses of reading times on the interest regions for older and younger adults in Experiment 2

Measure	Fixed effect	*b*	*SE*	*t*
Target − 1	Intercept	**5.86**	**0.02**	**252.55**
	Age	**0.54**	**0.05**	**11.72**
	Predictability	0.00	0.01	0.19
	Relatedness	− 0.00	0.01	− 0.42
	Length	**0.01**	**0.00**	**2.35**
	Frequency	− 0.00	0.00	− 0.46
	Pred target: Constraint	− 0.01	0.01	− 0.84
	Rel target: Constraint	− 0.01	0.01	− 0.77
	Unrel target: Constraint	− 0.01	0.01	− 0.90
	Age × Predictability	0.00	0.01	0.47
	Age × Relatedness	0.00	0.01	0.18
	Age × Pred target: Constraint	− 0.01	0.02	− 0.65
	Age × Rel target: Constraint	0.02	0.02	1.18
	Age × Unrel target: Constraint	− 0.02	0.02	− 1.13
Target	Intercept	**5.88**	**0.03**	**226.81**
	Age	**0.58**	**0.05**	**11.25**
	Predictability	− 0.01	0.01	− 1.36
	Relatedness	− 0.01	0.01	− 0.90
	Pred target: Constraint	0.01	0.01	0.51
	Rel target: Constraint	− 0.02	0.01	− 1.29
	Unrel target: Constraint	** − 0.03**	**0.01**	** − 2.68**
	Age × Predictability	0.00	0.01	0.23
	Age × Relatedness	0.00	0.01	0.24
	Age × Pred target: Constraint	0.02	0.02	0.85
	Age × Rel target: Constraint	0.01	0.02	0.62
	Age × Unrel target: Constraint	− 0.04	0.02	− 1.68
Target + 1	Intercept	**5.91**	**0.03**	**238.50**
	Age	**0.55**	**0.05**	**11.23**
	Predictability	** − 0.03**	**0.01**	** − 6.20**
	Relatedness	** − 0.02**	**0.01**	** − 2.61**
	Length	**0.01**	**0.00**	**3.04**
	Frequency	0.00	0.00	0.85
	Pred target: Constraint	**0.02**	**0.01**	**2.09**
	Rel target: Constraint	− 0.01	0.01	− 0.72
	Unrel target: Constraint	** − 0.06**	**0.01**	** − 4.30**
	Age × Predictability	− 0.00	0.01	− 0.02
	Age × Relatedness	0.01	0.01	0.64
	Age × Pred target: Constraint	− 0.00	0.02	− 0.19
	Age × Rel target: Constraint	− 0.01	0.02	− 0.42
	Age × Unrel target: Constraint	− 0.03	0.02	− 1.21

*Note.* Significant effects are bolded. Pred = predictable; Rel = related; Unrel = unrelated

The outcomes of the combined analyses averaged over age were virtually identical to the outcomes of the analyses restricted to older adults with one exception: There was a main effect of constraint on the word following predictable targets (i.e., Target + 1) because, averaged over age, reading times were shorter following these words in strong versus weak contexts (*t* = 2.09). Across all three interest regions, the main effect of age was only significant on overall reading times, which were slower for older compared with younger adults (|*t*|s > 11.23). Age did not interact significantly with the constraint effect for any target type (|*t*|s < 1.68). Thus, as illustrated in Fig. 2, both age groups generally showed equivalent reading times across all three interest regions.

### Discussion

Experiment 2 assessed older adults’ use of anticipatory prediction by investigating whether they show processing costs for unexpected input in an SPR task, which uses an unnatural presentation format while presenting the same stimuli as the previous eye-movement experiment.

There was some indication that the less natural format affected older adults’ use of contextual information during reading. In contrast to Experiment 1, older adults did not process predictable words more efficiently in strong contexts, despite the availability of contextual constraint, compared with weak contexts. This extended to related words, which were processed equivalently across strong and weak contexts, indicating that these items did not benefit from semantic overlap with the most expected completion. Despite the absence of robust processing benefits for expected and related input, older adults showed longer reading times for unpredictable words that were unrelated to the best completion in strong contexts that encouraged the preactivation of a more expected competitor. This processing cost also extended to the word following unrelated targets, capturing ongoing disruption even after readers had moved beyond the unexpected input. Thus, like Experiment 1, older adults do appear to be sensitive to the consequences of making an incorrect prediction. It should be noted though that because the SPR record captures only a single reading measure—the amount of time taken to process each word before a button press reveals the next word—it is possible that these effects also reflect the late consequences of integrating an item that is semantically incompatible with the broader discourse representation.

The current SPR findings are therefore partly compatible with the findings of previous behavioral experiments using normal and self-paced reading. Despite slower and more variable reading times across the entire sentence, older adults use contextual information during reading like their younger counterparts, as evidenced by the processing costs for unrelated completions in strong contexts. The supplemental combined analyses of older and younger adults’ data further revealed that the age groups did not appear to differ in their processing of any of the targets, despite the small differences in text format. However, there was evidence that, averaged over age, the word following predictable targets was processed faster in strong versus weak contexts, implying that there may have been a time lag between target word processing and readers’ button presses. The predictive processes engaged in normal reading thus appear to extend to self-paced reading when older adults have control over the presentation rate despite the word-by-word presentation format.

## General discussion

The present research aimed to investigate older adults’ use of prediction during reading. Although previous eye-movement studies have reported preserved or enhanced predictability effects with age (Andrews et al., 2022; Cheimariou et al., 2021; Choi et al., 2017; Kliegl et al., 2004; Rayner et al., 2006; Veldre et al., 2022b; Zhang et al., 2022), evidence of the immediate processing costs that would be expected to accompany prediction failure remains elusive in older adults and may depend on stimuli presentation method.

Across both experiments, older adults showed processing costs for unexpected input that disconfirmed a more expected completion; however, these effects were more pronounced in self-paced reading times than in eye movements. In the eye-movement data, unpredictable words that were unrelated to the highest cloze completion received slightly longer gaze durations in strong versus weak contexts. In the SPR tasks, these targets yielded longer reading times in strong contexts, not only at the target but also the following word. In both methodologies then, older adults appear to be sensitive to the consequences of prediction failure, suggesting that they have likely generated lexical predictions about upcoming text. A mismatch between their expectations and the word actually presented therefore leads to a small processing disruption that reflects the temporary suppression of the expected word (Federmeier et al., 2007; Kutas, 1993) and/or revision of an existing discourse representation so that the unexpected input can be processed thoroughly (Brothers et al., 2015; DeLong et al., 2014). Older adults’ use of prediction is consistent with the fact that linguistic experience via reading accumulates over one’s lifespan (Payne et al., 2012; Ryskin et al., 2020), allowing more precise and refined predictions to be generated with age.

Notably, prediction error costs did not extend to unpredictable words that were related to the best completion in either methodology. In the eye-movement data, related completions received early and late processing benefits in strong contexts, while in the SPR data, these completions were processed equivalently across both constraint conditions, incurring neither predictability benefits nor costs in strong contexts. These findings imply that older adults also generated graded predictions about upcoming text, which involve the partial preactivation of upcoming words based on their morphosyntactic, syntactic, and semantic attributes (Federmeier, 2022; Levy, 2008; Luke & Christianson, 2016; Staub, 2015; Staub et al., 2015). This strategy thus allows predictability effects to arise across a range of contextual constraints, including less predictable words, as evidenced in the present results by the processing benefits across early and late reading measures for related completions in strong contexts. The use of lexical and graded predictions in older adults is consistent with previous findings in younger adults (Federmeier, 2022), providing further evidence that predictive processes remain unchanged across the lifespan.

The current findings, particularly from the eye-movement experiment, differ from Andrews et al.’s (2022) study by demonstrating that older adults do show evidence of prediction error cost in a controlled experimental design. Before speculating on a potential explanation for this discrepancy, it is necessary to rule out potential alternative explanations for this effect. Firstly, the role of any differences in lexical characteristics, such as frequency and word length, can be eliminated because the current experiments always presented the same unrelated completions in both constraint conditions. It is also possible to exclude the role of any difference in semantic plausibility for unrelated completions, which was significant but negligible across strong and weak contexts (4.5 vs. 4.7 out 5)—like past studies (Frisson et al., 2017; Wong et al., 2024c). Instead, the discrepancy could reflect older adults’ sensitivity to contextual information in the broader linguistic environment (see Brothers et al., 2017, 2019; Dave et al., 2021; Wong et al., 2024c, for similar evidence in young adults). That is, when there are weak or sparse predictive cues in the linguistic environment, such as in the naturalistic texts used in Andrews et al.’s corpus-based study, older adults may prefer to generate multiple possible continuations which are less likely to incur processing costs when disconfirmed by unexpected input. However, when there are strong or more predictive cues in the linguistic environment, such as in the present experiments, where half of the sentences were strongly constraining and completed by the most expected completion a third of the time, older adults may favor the prediction of a specific lexical candidate which is more likely to be confirmed. This strategic modulation of predictive processes may be especially important with age to ensure optimization of limited cognitive resources for successful comprehension (Huettig & Mani, 2016; Pickering & Gambi, 2018; Wong et al., 2024a).

The current experiments may also provide useful insights into the discrepant findings between eye-movement and ERP studies regarding the impact of aging on predictive processes. ERP studies to date argue that older adults are less reliant on contextual information because they show reduced N400 effects and no late frontal positivity for prediction violations (see Payne & Silcox, 2019, for a review). However, ERP findings may not generalize to normal reading because they could reflect older adults’ inability to adapt effectively to the unnatural RSVP paradigm in which each word of a sentence appears one at a time in a central location at a fixed pace. Because it is not possible to investigate the online processes underlying RSVP reading via overt behavioral responses, the present research utilized the related SPR paradigm to investigate how the task demands associated with less natural stimuli presentation methods might impact predictive processes during reading. Indeed, older adults showed significant disruption across the entire sentence during SPR compared with normal reading; however, their target word processing patterns were similar across the two formats. These converging findings thus indicate that the word-by-word presentation format which restricts word skipping, re-reading, and parafoveal processes and which is common to both SPR and RSVP paradigms is unlikely to discourage older adults’ use of prediction during reading. Instead, the source of the decline in age-related predictive processes in ERP studies could be the other features of RSVP reading including the central word presentation which removes spatial information (e.g., see Milledge et al., 2023, for evidence of potential processing differences), and the fixed-pace presentation rate which removes control over the rate of input and displays each word for 400 to 1,000 ms (e.g., see Payne & Federmeier, 2017, for evidence of potential processing differences). These unnatural modifications may require older adults to allocate more cognitive resources to lower-level processes like word identification and/or lexical access to maintain an adequate level of comprehension, which could subsequently leave fewer resources available for higher-level processes like prediction.

Several other noteworthy procedural factors could contribute to the mixed evidence of predictability effects in older adults. Unlike natural reading, ERP studies require the suppression of eye movements during RSVP reading which may not only impose an additional cognitive load on older adults during word identification (Rayner & Morrison, 1981; Veldre et al., 2022a) or text comprehension processes (Castelhano & Muter, 2001; Rubin & Turano, 1992) but also impede their ability to use contextual information in a predictive manner. Moreover, unlike eye-movement studies, which typically include occasional questions to encourage deep comprehension, ERP studies include either no task or a secondary task, like a memory recognition test, that requires little comprehension (e.g., Federmeier & Kutas, 2005; Wlotko & Federmeier, 2012; but see DeLong et al., 2012). These differing comprehension demands may lead older adults in ERP studies to process text in a shallower, “good enough” manner (Ferreira et al., 2002) that does not draw upon higher-order processes like prediction (see Andrews & Veldre, 2021; Andrews et al., 2022; Dave et al., 2018; Payne et al., 2019; Radach et al., 2008, for evidence that comprehension demands modulate online processing). For example, an ERP study by Dave et al. (2018) in which older and younger adults were instructed to predict passage-final words and report the accuracy of their predictions found predictability benefits and costs in both age groups suggesting that, under certain explicit comprehension demands, older adults do predict upcoming words ahead of time and are sensitive to the consequences of prediction failure. Overall, then, predictive processes in older adults appear to be sensitive to unnatural presentation methods and further research using the co-registration of eye movements and EEG during reading could provide further insights into the source of the discrepancy in age-related predictability effects between methodologies (see Dimigen et al., 2011; Himmelstoss et al., 2020 for reviews).

Although evidence of predictability costs across both experiments attests to older adults’ use of prediction during online processing, evidence of the predictability benefit differed across the methodologies. In the eye-movement data, predictable completions yielded early and late processing benefits in strong contexts, reflecting facilitation arising from both predictive and integrative mechanisms. In the SPR data, however, predictable completions failed to yield any processing benefits in strong contexts, either at the target or the following word. This decreased sensitivity to the presence of confirmed predictions is unlikely to reflect a weak predictability manipulation—disconfirmed predictions (i.e., words replaced by unrelated completions) yielded immediate processing costs in the same task, while the same predictable completions elicited processing benefits in the eye-movement task. Instead, the absence of predictability benefits during self-paced reading could reflect an upper limit on older adults’ processing benefits for expected input, either due to the cognitive demands of the word-by-word presentation format or the physical requirements of making continuous button presses to progress through the sentence. Given that older adults were able to slow down their reading times in response to prediction failure, it appears that these characteristics of the SPR task only affected whether older adults were able to optimize predictive strategies and not whether they were able to benefit from them.^6^

Taken together, the processing patterns exhibited by older adults provide limited support for the idea that older adults show age-related changes in eye-movement control during reading. Compared with younger adults, older readers showed similar predictability benefits and costs, as well as overall lower skipping rates, suggesting that they may not be as dependent on a contextually based risky reading strategy as has been previously suggested (see Choi et al., 2017; Veldre et al., 2021, 2022b; Zhang et al., 2022, for similar conclusions).^7^ Instead, the current patterns of reading behavior are more compatible with an account in which older adults are more cautious readers due to declines in their visual acuity and other cognitive abilities (see Owsley, 2011; Verhaeghen, 2013, for reviews). Older adults who volunteer in research with financial reimbursement may also be more motivated compared with younger adults who participate for course credit.

The final contribution of the current research was to evaluate the SPR task given its benefit as a low-cost methodology that can be used for web-based data collection with diverse populations (Marsden et al., 2018). Although older adults showed evidence of prediction error costs across both eye movements and self-paced reading times, there were notable differences in their processing of other completions between the methodologies. Predictable completions in strong contexts yielded processing benefits in the eye-movement but not SPR task, possibly reflecting the latter’s cognitive and motor demands due to the unnatural presentation and/or response requirements. Related completions in strong contexts similarly elicited late integration benefits in the eye-movement task but little evidence of comparable benefits in the SPR task, suggesting that the latter may also be insensitive to the full range of reading behaviors linked to later processing stages, including re-reading and making regressions to previous parts of text, due to its word-by-word presentation format.^8^ These discrepancies suggest several limitations associated with the SPR task which, importantly, only yields a single reading measure, in contrast to the eye-movement task which derives reading times typically assumed to distinguish early and late processing stages (Clifton et al., 2007; Vasishth et al., 2013). The utility of SPR paradigms may therefore be restricted to investigations of processes that occur early in the time course of online language comprehension and that are robust to the cognitive and physical demands of the unnatural presentation and/or response method. Recording eye movements during natural reading is arguably the gold standard for examining the online processes underlying reading.

In conclusion, the present research extends what is known about predictive processes beyond that of skilled, young-adult readers. Older adults appear to make use of prediction during online processing, consistent with other language-related abilities that remain resilient to age-related decline. However, the current findings also suggest that older adults’ propensity to engage predictive processes may depend on a variety of factors including information in their broader linguistic environment and stimuli presentation method. This is consistent with accumulating evidence in young adults that predictive processes are not ubiquitous but rather context-dependent and determined by the availability of cognitive resources (Huettig & Mani, 2016; Pickering & Gambi, 2018; Wong et al., 2024a). Predictive processing then, regardless of age, may be an important but flexible strategy for successful online language comprehension.

## Supplementary Information

Below is the link to the electronic supplementary material.

Supplementary file1 (DOCX 37.9 KB)

## Data Availability

The experimental materials and de-identified data for the present research are publicly available at the Open Science Framework website (https://osf.io/w6594/).

## Footnotes

1 For completeness, analyses of Target - 1 and Target + 1 (which are reported in Experiment 2) were also conducted. Both regions revealed the constraint effect across different target conditions; however, these findings should be interpreted with caution given that the overall skipping rate was relatively high (> 50%). Tables S1–3 summarize the complete mean reading measures and model outputs.

2 The other target reading measures included in the analyses of the older adults’ data (i.e., total fixation duration and the probability of regressions into the target) were not analyzed because they may have been influenced by reading of the follow-up sentence in Wong et al.’s (2024c) data.

3 For completeness, age comparisons of Target - 1 and Target + 1 (which are reported in Experiment 2) were also conducted. Only the word preceding the target revealed that age interacted with the constraint effect, although this interaction was not observed consistently across reading measures or target conditions. Table S4 summarizes the complete model outputs.

4 Some further evidence that related and unrelated unpredictable targets appear to behave differently in strong versus weak contexts comes from using a continuous measure of similarity to explain the eye-movement behavior. Following Brothers et al. (2023), the semantic relatedness between unpredictable targets and the predictable target was calculated using the CBOW feature of the word2vec architecture (*M*_related_ = 0.40, *SD* = 0.13; *M*_unrelated_ = 0.24, *SD* = 0.13; see Mandera et al., 2017; https://www.pawelmandera.com/snaut-en, 300 dimensions, window size 6, UKWAC + subtitle corpus). A subsequent analysis on the subset of unpredictable words in strong contexts found that, after controlling for cloze probability, increasing semantic relatedness was associated with shorter total fixation durations, *b* = − 0.25, *SE* = 0.11, *t* = − 2.35, suggesting that while related targets were processed faster under conditions of strong constraint, unrelated targets were processed slower in the same contexts.

5 The data for a further seven participants were excluded due to technical errors.

6 The distributions of the response time data across the three target conditions were all positively skewed, with unrelated words also showing a slightly more pronounced rightward tail. This pattern of distributions suggests that while all targets were processed relatively quickly, the processing of predictable targets was not selectively different from either of the unpredictable targets. The average duration of the button presses on the predictable targets were also similar to the average button-press duration on the other words of the sentence. Response times in strongly constraining contexts were 512 ms (*SD* = 181) for the predictable target and 517 ms (*SD* = 156) for all other words. Response times in weakly constraining contexts were 525 ms (*SD* = 201) for the predictable target and 520 ms (*SD* = 151) for all other words.

7 Supplementary analyses comparing global eye-movement measures for the age groups did reveal slower reading times across the entire sentence for older than younger adults due to a higher number of fixations and regressions and longer forward saccades, but no difference in skipping rates. The differences, however, could, in part, reflect the different text formats presented to the age groups (Radach et al., 2008)—the two-sentence passages presented to younger adults may have led to quicker first-pass reading of the initial sentence compared with the single sentences presented to older adults.

8 Further evidence of potential differences between the methodologies comes from the regions before and after the target. In particular, the word before the target appeared to be sensitive to the effects of constraint and age in the eye-tracking but not SPR data. Both regions also showed the constraint benefit for predictable targets on late eye-tracking measures, which did not extend to SPR measures. Notably, however, in both methodologies, the word following the target revealed evidence of spillover prediction error costs.

## Code availability

The analysis codes for the present research are publicly available at the Open Science Framework website (https://osf.io/w6594/).
