# Supplementary material for: Anticipatory prediction in older readers
Source: Mem Cognit. 2025 May 6;53(7):2312–31. doi: 10.3758/s13421-025-01712-1 (PMC12589329; doi:10.3758/s13421-025-01712-1)
Supplement: Supplementary file 1 — Supplementary file1 (DOCX 37.9 KB) [file 13421_2025_1712_MOESM1_ESM.docx]

Table S1
Mean (and standard deviation) Target−1 reading measures by condition in Experiment 1

|  | Predictable | | Related | | Unrelated | |
| --- | --- | --- | --- | --- | --- | --- |
|  | Strong constraint | Weak constraint | Strong constraint | Weak constraint | Strong constraint | Weak constraint |
| Skipping (%) | 0.51 (0.10) | 0.48 (0.09) | 0.50 (0.10) | 0.48 (0.11) | 0.53 (0.10) | 0.48 (0.11) |
| First fixation (ms) | 210 (19) | 202 (20) | 216 (17) | 198 (14) | 215 (21) | 202 (23) |
| Gaze (ms) | 226 (25) | 216 (25) | 240 (25) | 212 (23) | 237 (24) | 215 (26) |
| Total fixation (ms) | 263 (32) | 279 (47) | 286 (38) | 276 (34) | 326 (49) | 292 (39) |
| Regressions-out (%) | 0.07 (0.06) | 0.08 (0.07) | 0.06 (0.05) | 0.10 (0.06) | 0.06 (0.06) | 0.09 (0.06) |
| Regressions-in (%) | 0.14 (0.08) | 0.21 (0.09) | 0.19 (0.08) | 0.24 (0.10) | 0.25 (0.11) | 0.22 (0.12) |

Table S2
Mean (and standard deviation) Target+1 reading measures by condition in Experiment 1

|  | Predictable | | Related | | Unrelated | |
| --- | --- | --- | --- | --- | --- | --- |
|  | Strong constraint | Weak constraint | Strong constraint | Weak constraint | Strong constraint | Weak constraint |
| Skipping (%) | 0.53 (0.12) | 0.54 (0.10) | 0.49 (0.10) | 0.55 (0.09) | 0.48 (0.11) | 0.51 (0.10) |
| First fixation (ms) | 200 (18) | 206 (22) | 214 (18) | 217 (25) | 213 (22) | 213 (24) |
| Gaze (ms) | 211 (31) | 222 (30) | 233 (28) | 237 (29) | 232 (28) | 228 (24) |
| Total fixation (ms) | 245 (43) | 288 (79) | 287 (58) | 309 (76) | 311 (50) | 286 (54) |
| Regressions-out (%) | 0.05 (0.06) | 0.06 (0.05) | 0.09 (0.06) | 0.09 (0.06) | 0.11 (0.07) | 0.08 (0.05) |
| Regressions-in (%) | 0.13 (0.10) | 0.19 (0.09) | 0.15 (0.09) | 0.19 (0.10) | 0.17 (0.08) | 0.20 (0.10) |

Table S3

LMM summaries for analyses of Target−1 and Target+1 reading measures in Experiment 1

|  |  | *Target−1* | | |  | *Target+1* | | |
| --- | --- | --- | --- | --- | --- | --- | --- | --- |
| Measure | Fixed effect | *b* | *SE* | *t*/*z* |  | *b* | *SE* | *t*/*z* |
| Skipping | Intercept | -0.11 | 0.09 | -1.15 |  | -0.06 | 0.09 | -0.58 |
|  | Predictability | -0.04 | 0.07 | -0.59 |  | **0.16** | **0.07** | **2.35** |
|  | Relatedness | -0.08 | 0.08 | -1.04 |  | 0.13 | 0.08 | 1.70 |
|  | Length | **-0.05** | **0.04** | **-10.87** |  | **-0.50** | **0.04** | **-11.98** |
|  | Frequency | **0.10** | **0.03** | **3.64** |  | **0.163** | **0.03** | **4.93** |
|  | Pred target: Constraint | **-0.50** | **0.14** | **-3.48** |  | -0.15 | 0.13 | -1.14 |
|  | Rel target: Constraint | **-0.43** | **0.16** | **-2.79** |  | 0.12 | 0.11 | 1.03 |
|  | Unrel target: Constraint | **-0.65** | **0.16** | **-4.02** |  | -0.00 | 0.113 | -0.04 |
|  |  |  |  |  |  |  |  |  |
| First fixation | Intercept | **5.27** | **0.02** | **242.87** |  | 5.26 | 0.03 | 201.53 |
|  | Predictability | -0.01 | 0.01 | -0.54 |  | **-0.05** | **0.01** | **-4.25** |
|  | Relatedness | -0.00 | 0.01 | -0.13 |  | 0.01 | 0.01 | 0.67 |
|  | Length | 0.00 | 0.00 | 0.16 |  | 0.00 | 0.01 | 0.70 |
|  | Frequency | **-0.01** | **0.00** | **-2.64** |  | **-0.02** | **0.01** | **-3.12** |
|  | Pred target: Constraint | -0.03 | 0.03 | -1.17 |  | 0.03 | 0.02 | 1.43 |
|  | Rel target: Constraint | **-0.06** | **0.02** | **-3.32** |  | 0.00 | 0.02 | 0.19 |
|  | Unrel target: Constraint | -0.04 | 0.02 | -1.68 |  | -0.01 | 0.03 | -0.38 |
|  |  |  |  |  |  |  |  |  |
| Gaze | Intercept | **5.32** | **0.03** | **212.12** |  | **5.29** | **0.03** | **181.66** |
|  | Predictability | -0.02 | 0.01 | -1.37 |  | **-0.07** | **0.01** | **-4.95** |
|  | Relatedness | 0.01 | 0.01 | 0.45 |  | 0.02 | 0.02 | 1.38 |
|  | Length | **0.01** | **0.01** | **2.32** |  | 0.01 | 0.01 | 1.64 |
|  | Frequency | **-0.01** | **0.00** | **-3.98** |  | **-0.03** | **0.01** | **-4.60** |
|  | Pred target: Constraint | -0.01 | 0.03 | -0.41 |  | 0.04 | 0.03 | 1.70 |
|  | Rel target: Constraint | **-0.07** | **0.02** | **-3.06** |  | 0.01 | 0.03 | 0.35 |
|  | Unrel target: Constraint | -0.04 | 0.02 | -1.49 |  | -0.04 | 0.03 | -1.33 |
|  |  |  |  |  |  |  |  |  |
| Total fixation | Intercept | **5.47** | **0.03** | **158.26** |  | **5.43** | **0.03** | **160.76** |
|  | Predictability | **-0.07** | **0.02** | **-4.49** |  | **-0.11** | **0.02** | **-6.48** |
|  | Relatedness | **-0.06** | **0.02** | **-3.42** |  | -0.01 | 0.02 | -0.56 |
|  | Length | **0.03** | **0.01** | **4.33** |  | **0.05** | **0.01** | **5.15** |
|  | Frequency | **-0.02** | **0.01** | **-4.41** |  | **-0.04** | **0.01** | **-5.46** |
|  | Pred target: Constraint | **0.11** | **0.03** | **3.64** |  | **0.12** | **0.03** | **4.40** |
|  | Rel target: Constraint | 0.04 | 0.03 | 1.31 |  | 0.03 | 0.04 | 1.00 |
|  | Unrel target: Constraint | -0.01 | 0.04 | -0.18 |  | **-0.08** | **0.04** | **-2.30** |
|  |  |  |  |  |  |  |  |  |
| Regressions-out | Intercept | **-2.79** | **0.14** | **-19.81** |  | **-2.81** | **0.13** | **-22.16** |
|  | Predictability | 0.02 | 0.11 | 0.18 |  | **-0.74** | **0.13** | **-5.62** |
|  | Relatedness | 0.04 | 0.13 | 0.28 |  | -0.07 | 0.12 | -0.55 |
|  | Length | **0.12** | **0.04** | **2.79** |  | **0.31** | **0.06** | **4.88** |
|  | Frequency | -0.05 | 0.03 | -1.59 |  | 0.05 | 0.05 | 0.92 |
|  | Pred target: Constraint | 0.41 | 0.23 | 1.82 |  | 0.28 | 0.30 | 0.96 |
|  | Rel target: Constraint | **0.76** | **0.19** | **3.98** |  | 0.08 | 0.24 | 0.33 |
|  | Unrel target: Constraint | **0.69** | **0.19** | **3.59** |  | -0.28 | 0.18 | -1.58 |
|  |  |  |  |  |  |  |  |  |
| Regressions-in | Intercept | **-1.50** | **0.11** | **-13.43** |  | **-1.78** | **0.12** | **-14.50** |
|  | Predictability | **-0.32** | **0.08** | **-4.16** |  | **-0.19** | **0.08** | **-2.29** |
|  | Relatedness | -0.12 | 0.08 | -1.40 |  | -0.13 | 0.09 | -1.46 |
|  | Length | -0.02 | 0.04 | -0.50 |  | **0.10** | **0.05** | **2.09** |
|  | Frequency | **-0.09** | **0.02** | **-3.85** |  | -0.05 | 0.04 | -1.38 |
|  | Pred target: Constraint | **0.69** | **0.15** | **4.73** |  | **0.56** | **0.16** | **3.39** |
|  | Rel target: Constraint | **0.52** | **0.17** | **3.16** |  | 0.29 | 0.18 | 1.57 |
|  | Unrel target: Constraint | 0.00 | 0.19 | 0.01 |  | 0.26 | 0.15 | 1.71 |

Note. Significant effects are bolded.

Table S4

LMM summaries for analyses of Target−1 and Target+1 reading measures for older and younger adults in Experiment 1

|  |  | *Target−1* | | |  | *Target+1* | | |
| --- | --- | --- | --- | --- | --- | --- | --- | --- |
| Measure | Fixed effect | *b* | *SE* | *t*/*z* |  | *b* | *SE* | *t*/*z* |
| Skipping | Intercept | 0.01 | 0.07 | 0.18 |  | **0.14** | **0.06** | **2.39** |
|  | Age | -0.16 | 0.10 | -1.62 |  | -0.15 | 0.10 | -1.49 |
|  | Predictability | -0.04 | 0.04 | -0.85 |  | **0.14** | **0.04** | **3.20** |
|  | Relatedness | -0.02 | 0.05 | -0.37 |  | 0.10 | 0.05 | 1.95 |
|  | Length | **-0.38** | **0.03** | **-13.15** |  | **-0.43** | **0.03** | **-13.30** |
|  | Frequency | **0.13** | **0.02** | **7.09** |  | **0.14** | **0.02** | **5.56** |
|  | Pred target: Constraint | **-0.36** | **0.10** | **-3.59** |  | -0.04 | 0.08 | -0.43 |
|  | Rel target: Constraint | **-0.29** | **0.12** | **-2.35** |  | 0.14 | 0.09 | 1.47 |
|  | Unrel target: Constraint | **-0.38** | **0.11** | **-3.43** |  | 0.08 | 0.09 | 0.91 |
|  | Age*Predictability | 0.00 | 0.09 | 0.01 |  | 0.01 | 0.09 | 0.16 |
|  | Age*Relatedness | -0.09 | 0.10 | -0.93 |  | 0.05 | 0.10 | 0.51 |
|  | Age*Pred target: Constraint | **-0.30** | **0.15** | **-2.04** |  | -0.14 | 0.14 | -0.94 |
|  | Age*Rel target: Constraint | **-0.36** | **0.14** | **-2.46** |  | 0.02 | 0.15 | 0.11 |
|  | Age*Unrel target: Constraint | **-0.52** | **0.14** | **-3.64** |  | -0.11 | 0.14 | -0.75 |
|  |  |  |  |  |  |  |  |  |
| First fixation | Intercept | **5.28** | **0.01** | **359.07** |  | 5.27 | **0.02** | **322.10** |
|  | Age | **-0.01** | **0.03** | **-0.43** |  | -0.02 | 0.03 | -0.69 |
|  | Predictability | -0.01 | 0.01 | -1.28 |  | **-0.06** | **0.01** | **-6.37** |
|  | Relatedness | -0.01 | 0.01 | -0.81 |  | 0.01 | 0.01 | 0.80 |
|  | Length | -0.00 | 0.00 | -1.05 |  | 0.01 | 0.01 | 1.06 |
|  | Frequency | **-0.01** | **0.00** | **-3.60** |  | **-0.01** | **0.00** | **-3.49** |
|  | Pred target: Constraint | -0.02 | 0.02 | -1.18 |  | **0.05** | **0.02** | **2.81** |
|  | Rel target: Constraint | **-0.04** | **0.02** | **-2.34** |  | 0.02 | 0.02 | 0.84 |
|  | Unrel target: Constraint | **-0.07** | **0.02** | **-3.60** |  | -0.00 | 0.01 | -0.19 |
|  | Age*Predictability | 0.01 | 0.02 | 0.48 |  | 0.00 | 0.02 | 0.05 |
|  | Age*Relatedness | 0.01 | 0.02 | 0.57 |  | 0.01 | 0.02 | 0.05 |
|  | Age*Pred target: Constraint | -0.02 | 0.03 | -0.64 |  | -0.03 | 0.03 | -0.91 |
|  | Age*Rel target: Constraint | -0.05 | 0.03 | -1.84 |  | -0.02 | 0.03 | -0.61 |
|  | Age*Unrel target: Constraint | 0.06 | 0.03 | 1.88 |  | -0.01 | 0.03 | -0.44 |
|  |  |  |  |  |  |  |  |  |
| Gaze | Intercept | **5.32** | **0.02** | **314.53** |  | **5.30** | **0.02** | **288.42** |
|  | Age | -0.01 | 0.03 | -0.32 |  | -0.02 | 0.04 | -0.67 |
|  | Predictability | -0.02 | 0.01 | -1.94 |  | **-0.07** | **0.01** | **-7.40** |
|  | Relatedness | -0.01 | 0.01 | -0.78 |  | 0.01 | 0.01 | 1.33 |
|  | Length | **0.01** | **0.00** | **2.66** |  | **0.02** | **0.01** | **2.52** |
|  | Frequency | **-0.01** | **0.00** | **-4.41** |  | **-0.02** | **0.00** | **-4.68** |
|  | Pred target: Constraint | 0.001 | 0.02 | 0.03 |  | **0.05** | **0.02** | **2.83** |
|  | Rel target: Constraint | -0.03 | 0.02 | -1.38 |  | 0.02 | 0.02 | 0.81 |
|  | Unrel target: Constraint | **-0.05** | **0.02** | **-2.79** |  | -0.01 | 0.02 | -0.62 |
|  | Age*Predictability | -0.00 | 0.02 | -0.03 |  | 0.00 | 0.02 | 0.16 |
|  | Age*Relatedness | 0.03 | 0.02 | 1.37 |  | 0.01 | 0.02 | 0.50 |
|  | Age*Pred target: Constraint | -0.03 | 0.03 | -1.11 |  | -0.01 | 0.03 | -0.37 |
|  | Age*Rel target: Constraint | **-0.09** | **0.03** | **-2.70** |  | -0.01 | 0.04 | -0.27 |
|  | Age*Unrel target: Constraint | 0.02 | 0.03 | 0.74 |  | -0.04 | 0.03 | -1.42 |
|  |  |  |  |  |  |  |  |  |
| Regressions-out | Intercept | **-2.67** | **0.10** | **-27.52** |  | **-2.71** | **0.08** | **-33.08** |
|  | Age | -0.28 | 0.17 | -1.70 |  | -0.22 | 0.14 | -1.51 |
|  | Predictability | 0.06 | 0.07 | 0.77 |  | **-0.45** | **0.08** | **-5.47** |
|  | Relatedness | 0.01 | 0.08 | 0.07 |  | -0.13 | 0.08 | -1.59 |
|  | Length | **0.09** | **0.03** | **2.76** |  | **0.26** | **0.05** | **5.28** |
|  | Frequency | **-0.07** | **0.02** | **-3.14** |  | 0.01 | 0.04 | 0.25 |
|  | Pred target: Constraint | **0.31** | **0.15** | **2.01** |  | 0.34 | 0.19 | 1.84 |
|  | Rel target: Constraint | **0.37** | **0.17** | **2.19** |  | 0.09 | 0.19 | 0.48 |
|  | Unrel target: Constraint | **0.34** | **0.14** | **2.50** |  | -0.23 | 0.14 | -1.56 |
|  | Age*Predictability | -0.04 | 0.14 | -0.31 |  | **-0.48** | **0.16** | **-3.05** |
|  | Age*Relatedness | 0.01 | 0.17 | 0.06 |  | 0.11 | 0.16 | 0.71 |
|  | Age*Pred target: Constraint | 0.28 | 0.23 | 1.22 |  | -0.16 | 0.28 | -0.57 |
|  | Age*Rel target: Constraint | **0.83** | **0.26** | **3.21** |  | -0.08 | 0.26 | -0.29 |
|  | Age*Unrel target: Constraint | **0.77** | **0.24** | **3.23** |  | -0.10 | 0.24 | -0.42 |

Note. Significant effects are bolded. Younger adults (n=57) from Wong et al. (2024c; Experiment 2).

**References:**

Wong, R., Veldre, A., & Andrews, S. (2024c). Looking downstream for evidence of lexical prediction in eye movements during reading. Quarterly Journal of Experimental Psychology, 77(10), 2040-2064. https://doi.org/10.1177/17470218231223858.
